# Supplementary material for: Profiles of lipid, protein and microRNA expression in exosomes derived from intestinal epithelial cells after ischemia-reperfusion injury in a cellular hypoxia model
Source: PLoS One. 2023 Mar 29;18(3):e0283702. doi: 10.1371/journal.pone.0283702 (PMC10058167; doi:10.1371/journal.pone.0283702)
Supplement: S1 Table — The annotation was obtained from the UniProt database (https://www.uniprot.org/). (DOCX) [file pone.0283702.s001.docx]

**Supplementary Table S1.**

Annotation of proteins related to inflammation in Gene Ontology (GO) terms. The annotation was obtained from the UniProt database (<https://www.uniprot.org/>).

| **Protein** | **Annotation** |
| --- | --- |
| **PYGL** | Glycogen phosphorylase, liver form. |
| **PYGB** | Glycogen phosphorylase, brain form; glycogen phosphorylase regulates glycogen mobilization. |
| **NAMPT** | Nicotinamide phosphoribosyltransferase; catalyzes the condensation of nicotinamide with 5-phosphoribosyl-1-pyrophosphate to yield nicotinamide mononucleotide, an intermediate in the biosynthesis of NAD. |
| **C3** | Complement C3; C3 plays a central role in the activation of the complement system. Its generation using C3 convertase is the central reaction in both classical and alternative complement pathways. |
| **ACLY** | ATP-citrate synthase; ATP-citrate synthase is the primary enzyme responsible for the synthesis of cytosolic acetyl-CoA in many tissues. It plays a key role in *de novo* lipid synthesis. |
| **PSMD3** | 26S proteasome non-ATPase regulatory subunit 3; component of the 26S proteasome, a multiprotein complex involved in the ATP-dependent degradation of ubiquitinated proteins. |
| **AGL** | Glycogen debranching enzyme; multifunctional enzyme acting as 1,4-alpha-D-glucan:1,4-alpha-D-glucan 4-alpha-D-glycosyltransferase and amylo-1,6-glucosidase in glycogen degradation. |
| **DYNC1H1** | Cytoplasmic dynein 1 heavy chain 1; cytoplasmic dynein 1 acts as a motor for the intracellular retrograde motility of vesicles and organelles along microtubules. |
| **XRCC6** | X-ray repair cross-complementing protein 6; single-stranded DNA-dependent ATP-dependent helicase. It has a role in chromosome translocation. |
| **UBR4** | E3 ubiquitin-protein ligase UBR4; E3 ubiquitin-protein ligase, which is a component of the N-end rule pathway. It recognizes and binds to proteins bearing specific N-terminal residues that are destabilizing according to the N-end rule, leading to their ubiquitination and subsequent degradation. |
| **DDX3X** | ATP-dependent RNA helicase DDX3X; multifunctional ATP-dependent RNA helicase. The ATPase activity can be stimulated by various ribonucleic and deoxynucleic acids indicative of relaxed substrate specificity. |
| **PSMC2** | 26S proteasome regulatory subunit 7; component of the 26S proteasome, a multiprotein complex involved in the ATP-dependent degradation of ubiquitinated proteins. |
| **CAPN1** | Calpain-1 catalytic subunit; calcium-regulated non-lysosomal thiol-protease that catalyzes limited proteolysis of substrates involved in cytoskeletal remodeling and signal transduction; belongs to the peptidase C2 family. |
| **CAND1** | Cullin-associated NEDD8-dissociated protein 1; key assembly factor of SCF (SKP1-CUL1-F-box protein)–E3 ubiquitin ligase complexes that promotes the exchange of the substrate-recognition F-box subunit in SCF complexes, thereby playing a pivotal role in the cellular repertoire of SCF complexes. |
| **CYFIP1** | Cytoplasmic FMR1-interacting protein 1; a component of the CYFIP1-EIF4E-FMR1 complex that binds to the mRNA cap and mediates translational repression. In the CYFIP1-EIF4E-FMR1 complex, this subunit is an adapter between EIF4E and FMR1. |
