# Supplementary figures and images for: Profiles of lipid, protein and microRNA expression in exosomes derived from intestinal epithelial cells after ischemia-reperfusion injury in a cellular hypoxia model
Source: PLoS One. 2023 Mar 29;18(3):e0283702. doi: 10.1371/journal.pone.0283702 (PMC10058167; doi:10.1371/journal.pone.0283702)

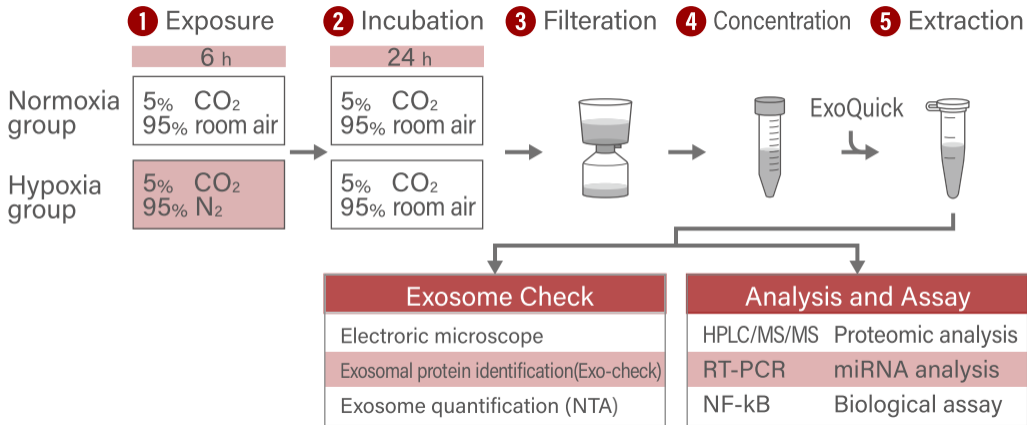

Supplement: S1 Fig — (PDF) [file pone.0283702.s002.pdf]

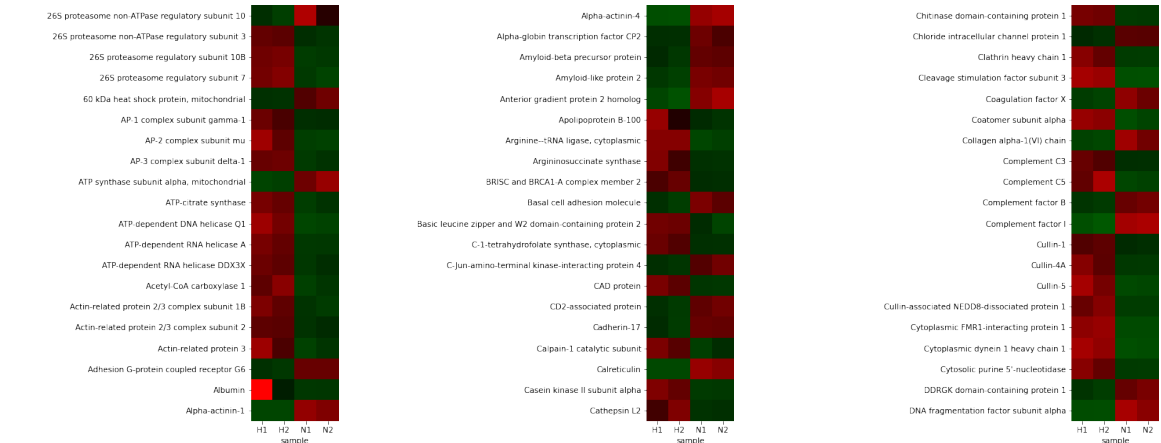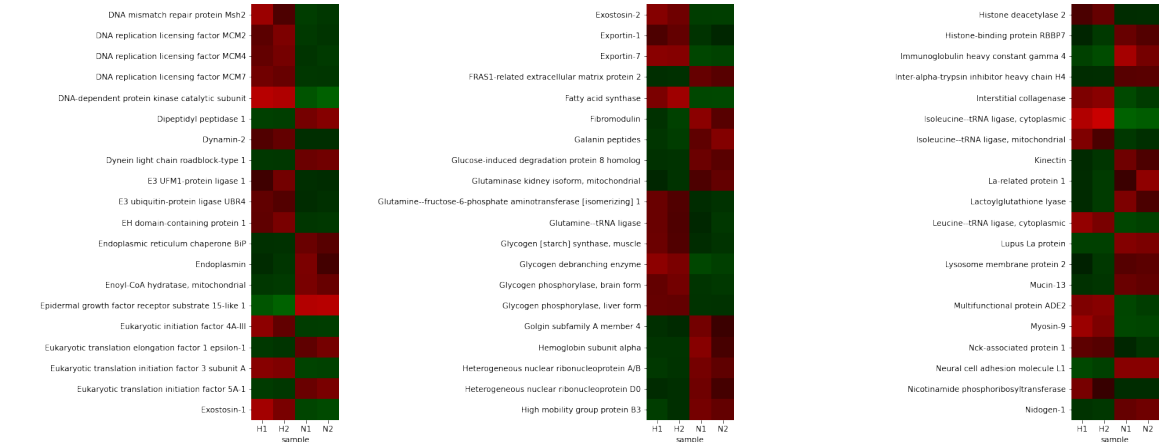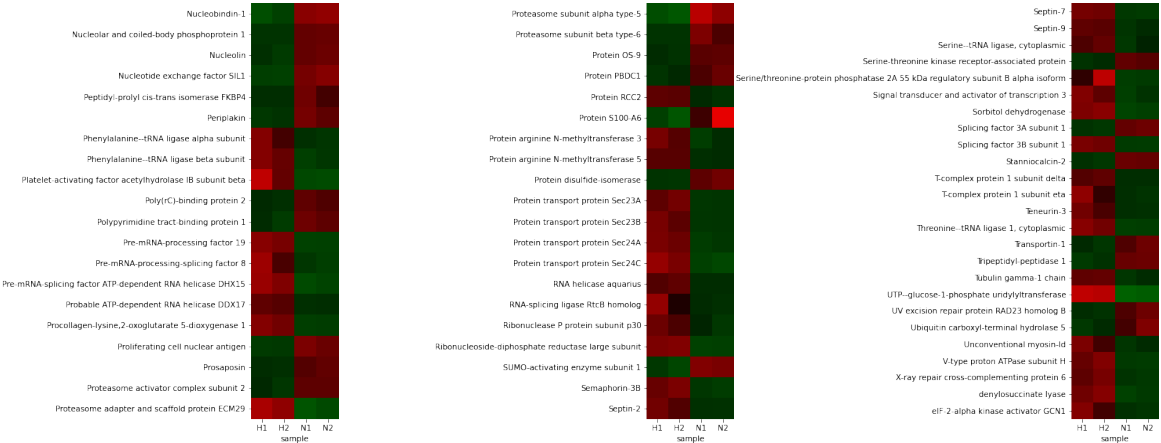

Supplement: S2 Fig — (PDF) [file pone.0283702.s003.pdf]
