# Supplementary material for: Profiles of lipid, protein and microRNA expression in exosomes derived from intestinal epithelial cells after ischemia-reperfusion injury in a cellular hypoxia model
Source: PLoS One. 2023 Mar 29;18(3):e0283702. doi: 10.1371/journal.pone.0283702 (PMC10058167; doi:10.1371/journal.pone.0283702)
Supplement: S1 Methods — (DOCX) [file pone.0283702.s004.docx]

**Supplementary Methods S1**

**List of primers used in the miRNA PCR array**

hsa-miR-142-5p, hsa-miR-9-5p, hsa-miR-150-5p, hsa-miR-27b-3p, hsa-miR-101-3p, hsa-let-7d-5p, hsa-miR-103a-3, hsa-miR-16-5p, hsa-miR-26a-5p, hsa-miR-32-5p, hsa-miR-26b-5p, hsa-let-7g-5p, hsa-miR-30c-5p, hsa-miR-96-5p, hsa-miR-185-5p, hsa-miR-142-3p, hsa-miR-24-3p, hsa-miR-155-5p, hsa-miR-146a-5, hsa-miR-425-5p, hsa-miR-181b-5, hsa-miR-302b-3, hsa-miR-30b-5p, hsa-miR-21-5p, hsa-miR-30e-5p, hsa-miR-200c-3, hsa-miR-15b-5p, hsa-miR-223-3p, hsa-miR-194-5p, hsa-miR-210, hsa-miR-15a-5p, hsa-miR-181a-5, hsa-miR-125b-5, hsa-miR-99a-5p, hsa-miR-28-5p, hsa-miR-320a, hsa-miR-125a-5, hsa-miR-29b-3p, hsa-miR-29a-3p, hsa-miR-141-3p, hsa-miR-19a-3p, hsa-miR-18a-5p, hsa-miR-374a-5, hsa-miR-423-5p, hsa-let-7a-5p, hsa-miR-124-3p, hsa-miR-92a-3p, hsa-miR-23a-3p, hsa-miR-25-3p, hsa-let-7e-5p, hsa-miR-376c-3, hsa-miR-126-3p, hsa-miR-144-3p, hsa-miR-424-5p, hsa-miR-30a-5p, hsa-miR-23b-3p, hsa-miR-151a-5, hsa-miR-195-5p, hsa-miR-143-3p, hsa-miR-30d-5p, hsa-miR-191-5p, hsa-let-7i-5p, hsa-miR-302a-3, hsa-miR-222-3p, hsa-let-7b-5p, hsa-miR-19b-3p, hsa-miR-17-5p, hsa-miR-93-5p, hsa-miR-186-5p hsa-miR-196b-5, hsa-miR-27a-3p, hsa-miR-22-3p, hsa-miR-130a-3,hsa-let-7c,hsa-miR-29c-3p,hsa-miR-140-3p,hsa-miR-128, hsa-let-7f-5p,hsa-miR-122-5p,hsa-miR-20a-5p,hsa-miR-106b-5, hsa-miR-7-5p, hsa-miR-100-5p, and hsa-miR-302c-3.
